# The underlying structure of the English Cancer Patient Experience Survey: Factor analysis to support survey reporting and design

**DOI:** 10.1002/cam4.4325

**Published:** 2021-12-05

**Authors:** Mayam Gomez‐Cano, Georgios Lyratzopoulos, John L. Campbell, Marc N. Elliott, Gary A. Abel

**Affiliations:** ^1^ University of Exeter Medical School (Primary Care) University of Exeter Exeter UK; ^2^ Epidemiology of Cancer Healthcare and Outcomes (ECHO) Group Department of Behavioural Science and Health University College London London UK; ^3^ RAND Corporation Santa Monica California USA

**Keywords:** cancer, factor analysis, health services administration, indicators, oncology, patient experience, psycho‐oncology, survey, surveys and questionnaires

## Abstract

**Background:**

The English Cancer Patient Experience Survey (CPES) is a regularly conducted survey measuring the experience of cancer patients. We studied the survey's underlying structure using factor analysis to identify potential for improvements in reporting or questionnaire design.

**Methods:**

Cancer Patient Experience Survey 2015 respondents (*n* = 71,186, response rate 66%) were split into two random subgroups. Using exploratory factor analysis (EFA) on the first subgroup, we identified the survey's latent structure. EFA was then applied to 12 sets of items. A first (“core”) set was formed by questions that applied to all participants. The subsequent sets contained the “core set” plus questions corresponding to specific care pathways/patient groups. We used confirmatory factor analysis (CFA) on the second data subgroup for cross‐validation.

**Results:**

The EFA suggested that five latent factors underlie the survey's core questions. Analysis on the remaining 11 care pathway/patient group items also indicated the same five latent factors, although additional factors were present for questions applicable to patients with an overnight stay or those accessing specialist nursing. The five factors models had an excellent fit (comparative fit index = 0.95, root mean square error of approximation = 0.045 for core set of questions). Items loading on each factor generally corresponded to a specific section or subsection of the questionnaire. CFA findings were concordant with the EFA patterns.

**Conclusion:**

The findings suggest five coherent underlying sub‐constructs relating to different aspects of cancer health care. The findings support the construction of evidence‐based composite indicators for different domains of experience and provide options for survey re‐design.

## Graphical abstract

The underlying structure of the Cancer Patient experience Survey comprises five major factors. These include shared decisions; care coordination; diagnostic care; timely tests; and aftercare. Our results support the current survey structure, but provide options to guide survey redesign.

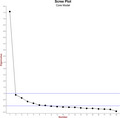

## 1 BACKGROUND

Patient experience has been established as a distinct domain of quality of care, together with clinical effectiveness and patient safety.^1^, ^2^, ^3^ Consequently, in recent decades modern healthcare systems conduct large patient surveys with nationwide coverage, whose findings are reported publicly for responsible/accountable organizations. Examples include the General Practice Patient Survey (GPPS) and the Adult Inpatient Survey in England, and the CAHPS surveys in the United States.^4^, ^5^, ^6^, ^7^ Although some such surveys encompass patients with any disease, some focus on the experience of patients with specific diseases. The English Cancer Patient Experience Survey (CPES) is an example of such a survey.^8^ To 2020, there have been eight waves of this survey from 2010 onward, with another two waves being prepared.

Ideally, the psychometric properties of survey questionnaires are examined during the survey design process. Often, as was the case with the CPES, surveys are implemented prior to any psychometric evaluation. In such cases, factor analysis can provide insights with a number of potential uses. Factor analysis is a family of statistical techniques which identify underlying, latent, relationships among survey items, helping to identify the constructs underpinning a survey. Using factor analysis, survey questions which relate to the same underlying construct or domain of care can be grouped together. These domains of care could be used as the basis for performance management and public reporting conventions. Organizations may be classified on the basis of their performance within each domain, rather than, or in addition to, being classified on every item. Knowledge of these domains might help to more efficiently target quality improvement efforts, addressing the source of deficits in patient experience, rather than each particular aspect of experience measured by individual questions.

Having identified domains, results of factor analysis can also inform future questionnaire development. The number of questions relating to the domain, and the consistency of responses to questions within that domain, can help to inform whether further questions are needed or if there is potential for item removal within a domain. Although the approach has been applied to other patient experience surveys,^9^, ^10^, ^11^, ^12^, ^13^, ^14^, ^15^, ^16^ no prior study has used factor analysis to identify the underlying structure of CPES. We therefore aimed to elucidate the structure of the CPES survey using factor analysis.

## 2 METHODS

### 2.1 Data

We used data from 71,186 respondents to the National CPES 2015 (response rate 65.7%). Details of the survey and method of administration have been published elsewhere.^17^ Briefly, the survey was mailed to all adult patients (aged 16 and over) discharged from a National Health Service hospital after inpatient or day case cancer‐related treatment during April–June 2015 following vital status checks at survey mail‐out (between 3 and 5 months after the sampling period).

The survey included 49 evaluative questions relating to aspects of patient experience (i.e., questions which ask patient to evaluate their care, which contrast with filter questions which often ask patients factual questions about their care to establish if a section of questions are relevant e.g., whether the patient has had an operation). It also includes questions about the patient (including age, gender, and ethnicity). Of the evaluative questions, seven have binary response options, 41 use a Likert scale with 3–7 response options, and one asks patients to rate their overall satisfaction between 0 and 10. Respondents were split randomly into two data sets, with one (*N* = 35,559) being used to establish the underlying structure of the data using exploratory factor analysis (EFA). The underlying factor structure was then confirmed using the second data set (*N* = 35,627) using confirmatory factor analysis (CFA).

### 2.2 Statistical analysis

#### 2.2.1 Core questions

Of the 49 evaluative questions, 20 represented domains of care that were assumed to be relevant to all respondents (with the remaining questions being relevant only to certain groups of patients, such as those treated by chemotherapy or those in education or employment––see below). We excluded one of these questions (relating to access to clinical nurse specialists) from the core set as it acts both as a measure of experience and a filter question. Throughout this work, we refer to these 19 items as “core questions.” Despite the high overall response rate of the survey, only 27% (19,263) of respondents gave an informative response to all the core questions (i.e., answers such as ‘Don't know/can't remember where treated as missing). Restricting analyses to this subset of respondents would result in reduced precision and the potential for bias that can arise from pairwise deletion.^18^ To counter this, we produced a single imputation of the missing responses using chained equations under the missing at random assumption. Predictive mean matching was used to maintain the interval nature of the data.

Reflecting the dichotomous and ordinal nature of the response options within CPES for most questions, we primarily employed categorical (polychoric) correlations within the EFA to avoid the attenuation of correlations between two categorical variables which can occur when Pearson correlations are used. We used linear (Pearson) correlations only for correlations involving the single 0–10 rating question. All correlations were computed using the *psych* package in R.^19^ We first performed an unrestricted EFA to determine the number of factors to retain. We used two methods for determining this number of factors: the Kaiser criterion, which identifies and retains factors with eigenvalues greater than one^20^ and the Cattell's scree test, which involves an examination of a plot of eigenvalues, the scree plot, for breaks or discontinuities.^21^ Having identified the number of factors, we performed additional EFA restricted to the number of factors identified by either method. We applied oblique rotations (using the *Promax* rotation method as implemented by the *psych* package in R^19^) when any of the two above methods indicated the retention of more than one factor with a view to explaining whether rotated models resulted in improved overall fit. These rotations lead to freely estimated inter‐factor correlations.^22^, ^23^ We use a cut‐off of 0.40 for the factor loadings.^24^ Items with lower loadings were removed.

To account for the ordinal nature of responses, the factor structures from the EFA were examined within the CFA using structural equations models applying Satorra–Bentler adjustment to the standard errors and chi‐squared values.^22^, ^23^ We made use of the population error statistic root mean square error of approximation (RMSEA), the baseline comparison statistics comparative fit index (CFI) and Tucker Lewis index (TLI), and the standardised root mean squared residuals (SRMR) statistic . The following cut‐off values are presently recognized as indicative of good fit: RMSE < 0.07, CFI ≥ 0.95, TLI ≥ 0.95, SRMR < 0.08.^25^, ^26^ CFA was performed using the *lavaan* package in R.^27^

The internal consistency reliability coefficients (Cronbach's alpha) for each factor derived from the EFA core model was computed using polychoric and Pearson correlations. The range of Cronbach's alpha coefficients in each factor when one question was left out was also calculated.

#### 2.2.2 Questions relating to specific patient groups/care pathways

Unlike “core” questions that every patient could have answered, most questions applied only to specific patient groups (e.g., those in education or employment) or to those who have undergone specific care pathways (e.g., having been treated by chemotherapy or having had an overnight stay in hospital). When responses to these questions were missing, it usually reflected the lack of applicability of a specific care pathway (i.e., patients without a hospital stay , therefore they should not answer questions regarding their experience as inpatients), rather than reflecting a lack of response to an applicable question. For this reason, we did not impute responses to questions relating to specific patient groups/care pathways and aspects of care.

Following previous work examining key drivers of satisfaction,^28^ we classified questions into 10 sets representing a specific patient group or care pathway, plus a further set including the question about access to clinical nurse specialists which was left out of the core set for analytic reasons (Appendix 1, Table A1). The above analysis for the core questions was repeated a further 11 times including responses to the core questions and responses to the questions applicable to the particular patient group/care pathway.

Analysis was performed using R 3.6.1.^29^

## 3 RESULTS

### 3.1 Core questions

**FIGURE 1:**
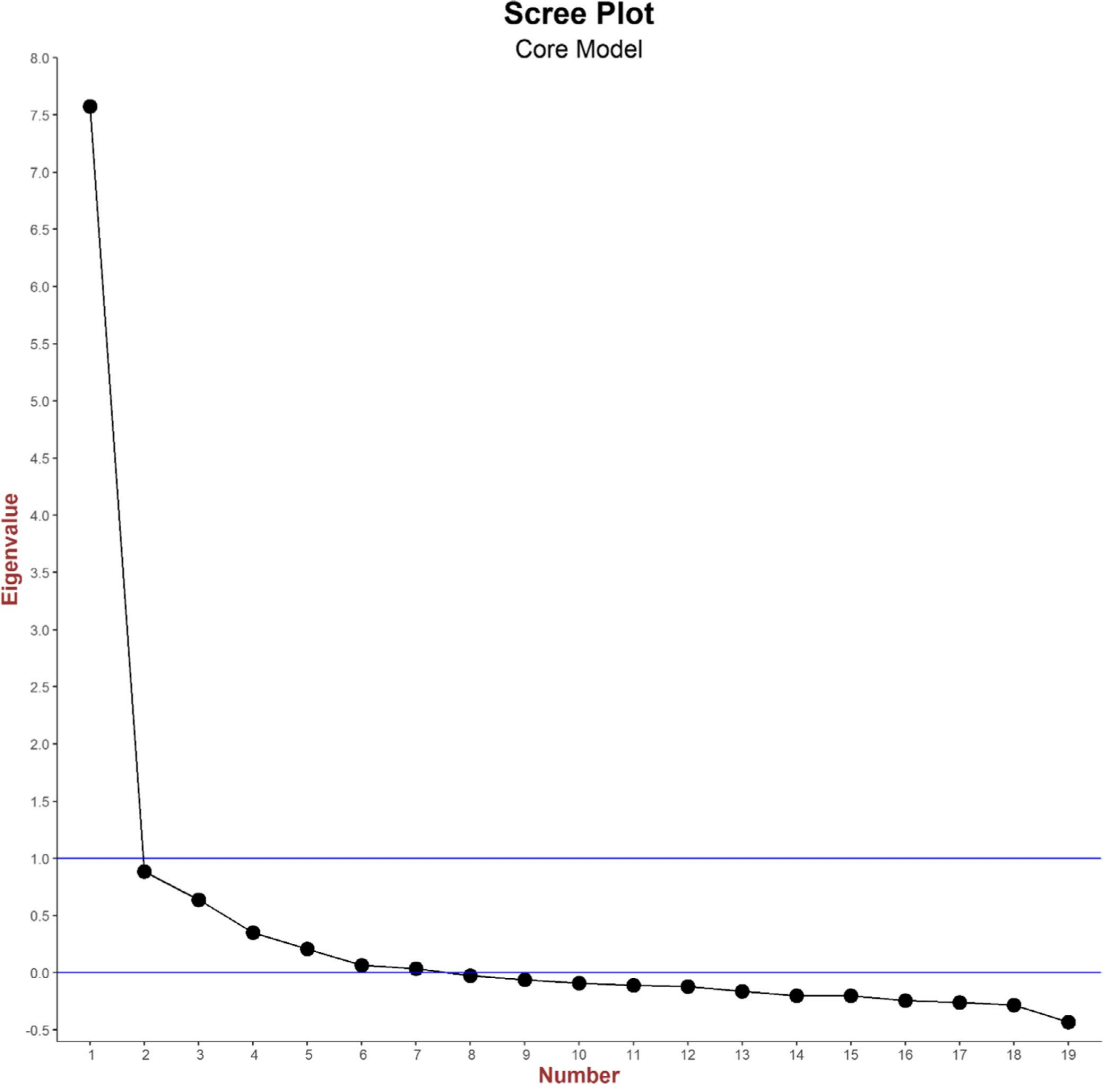
Scree plot for unrestricted exploratory factor analysis applied to the core set of 19 questions

The scree plot for unrestricted EFA applied to the core set of 19 questions which were applicable to all respondents is shown in Figure 1 indicating that only one factor had an eigenvalue > 1, suggesting a single unidimensional underlying patient experience construct for the data. A restricted EFA model with a single factor resulted in factor loadings > 0.4 for 18 of the 19 questions considered (Table 1). Only the question on willingness to take part in cancer research (Q58) had a loading < 0.4. Applying this one factor model (after removing Q58) within a CFA found that, depending on the goodness‐of‐fit measures that were used, the model did not provide a good fit to the data (RMSEA = 0.081, CFI = 0.836, and TLI = 0.814 indicating an unacceptable fit, and SRMR=0.054 indicating an acceptable fit––against recommended normative threshold values of RMSE < 0.07, CFI ≥ 0.95, TLI ≥ 0.95, and SRMR < 0.08).^25^, ^26^

**TABLE 1:** Factor matrix for exploratory factor analysis model restricted to a single factor and applied to the core set of 19 questions. Blanks correspond to loadings less than 0.4

Question number	Core model
	Factor 1
Q2	0.457
Q6	0.518
Q7	0.708
Q8	0.530
Q9	0.577
Q10	0.609
Q11	0.613
Q12	0.761
Q13	0.736
Q14	0.779
Q15	0.705
Q16	0.787
Q49	0.644
Q52	0.534
Q54	0.697
Q55	0.439
Q56	0.670
Q58	
Q59	0.742

We therefore examined the model structure implied by examination of the scree plot to determine the number of factors which should be retained. The scree plot (Figure 1) did not display any clear break or discontinuity. We therefore chose to retain five factors, corresponding to the point where the outstep decline ends (after factor five) and reaches a very low level (at factors five and six). Applying a five factor restricted EFA to the 19 core questions identified factor loadings > 0.4 for all questions, except for Q58 (Table 2). In general, the factors correspond to a domain or subdomain of care as explicitly captured by a section or subsection of the survey questionnaire. The questions loading on each factor are as follows:

- Factor 1: five items about treatment explanations and share decision‐making (Q12–Q16);
- Factor 2: four items about care coordination and administration (Q52, Q54, Q56, and Q59);
- Factor 3: five items about diagnosis (Q7–Q11);
- Factor 4: two items about timeliness of investigations (Q2 and Q6);
- Factor 5: two items about aftercare and support (Q49 and Q55).

**TABLE 2:** Factor matrix for exploratory factor analysis model restricted to five factors and applied to the core set of 19 questions. Blanks correspond to loadings less than 0.4

Question number	Core model				
	Factor 1	Factor 2	Factor 3	Factor 4	Factor 5
Q2				0.79512	
Q6				0.79354	
Q7			0.43189		
Q8			0.58687		
Q9			0.80632		
Q10			0.69871		
Q11			0.51439		
Q12	0.55				
Q13	0.91428				
Q14	0.7041				
Q15	0.72134				
Q16	0.5222				
Q49					0.4723
Q52		0.5632			
Q54		0.7609			
Q55					0.49
Q56		0.7783			
Q58					
Q59		0.86681			

Applying this five factor model within a CFA found that the model provided a good fit to the data for three of the four goodness‐of‐fit measures considered, with the fourth measure just below the acceptable threshold (RMSEA = 0.045 and SRMR = 0.029 indicating a good fit, CFI = 0.954 indicating an acceptable fit, and TLI = 0.944 just indicating an unacceptable fit).

The values of the Cronbach's alpha for each of the five factors fell within the acceptable 0.70 value with the exception of Factor 5, whose Cronbach's alpha was 0.60 (Appendix 1, Table A2). Deletion of one question from Factor 2 (“As far as you know, was your GP given enough information about your condition and the treatment you had at the hospital?”) led to an increase in Cronbach's alpha.

## 4 QUESTIONS RELATING TO SPECIFIC PATIENT GROUPS/CARE PATHWAYS

The scree plots for the unrestricted EFA models applied to the 11 sets of “patient group/pathway‐specific” questions (comprised of the core questions plus questions applicable to a particular patient group/care pathway) are shown in Appendix 2. When basing the number of factors to be retained on the basis of eigenvalues > 1 we retained only one factor in eight of the 11 patient group/pathway‐specific sets of questions. For the remaining three sets of questions, there are two eigenvalues > 1 (Appendix 2). As was the case with the core questions only model, in all 11 patient group/pathway‐specific EFA models restricted to one or two factors, the question on willingness to take part in cancer research (Q58) consistently had factor loadings < 0.4. This indicated that the question on willingness to take part in cancer research did not belong to either the core underlying construct of patient experience or the underlying construct of the additional patient group/pathway‐specific factor. For the 8/11 patient group/pathway‐specific sets of questions where (in restricted EFA) only one factor was retained, the noncore questions were all loaded (>0.4) onto this single factor, indicating they belonged to the core underlying construct of patient experience, with the exception of Q17 (“Were you given the name of a Clinical Nurse specialist?”). Where two factors were retained, the noncore questions all loaded onto a second factor defined by the noncore questions, namely;

- Questions about support for people with cancer.
- Questions about hospital stay.
- Questions about support from health and social care services outside hospital.

One or more core questions were loaded onto the new factor. No cross loadings were observed on any of these models.

As with models restricted to the core questions, applying a one or two factor CFA as appropriate to the question sets, did not provide a good fit to the data (see Appendix 1, Table A3).

As with the core set of questions, the scree plots for the 11 sets of questions (comprised of the core questions plus questions applicable to a particular patient group/care pathway) did not display any clear break or discontinuity. Instead, we retained a number of factors such that all factors present in the core questions only model were retained. In nine of the 11 patient group/pathway‐specific sets of questions this was achieved by retaining five factors. In two cases, an additional factor was retained (resulting in six factor models, see Table 3) which related to

- Questions about specialist nurse care.
- Questions about hospital stay.

**TABLE 3:** Loadings from the 11 five/six factor models corresponding to the 11 sets of questions defining 11 patient groups

Core questions			Noncore questions		
Q no.	Synoptic form	Loading	Q no.	Synoptic form	Loading
*Factor 1—Shared decision‐making*					
12	Treatment options explained	0.550^a^	44	Beforehand did you have all the information you needed for radiotx?	0.534^h^
13	Possible side effects of Tx explained/understandable?	0.914^a^	45	Were you given enough information about whether your radiotx was working?	0.449^h^
14	Practical advice and support in dealing with side effects of Tx	0.704^a^	47	Beforehand did you have all the information you needed for chemotx?	0.703^i^
15	Info on side effects of Tx in the longer term	0.721^a^	48	Were you given enough information about whether your chemotx was working?	0.525^i^
16	Were you involved in decisions about Tx as much as you wanted?	0.522^a^			
*Factor 2—Care coordination and administration*					
52	Did GP had enough info?	0.563^a^	38	Clear written information about what you should do or should not do after discharge	0.699^f^
54	Different people treating and caring for you work well together	0.761^a^	39	Were you told who to contact after leaving hospital if needed?	0.683^f^
56	Overall, how would you rate the administration of your care?	0.778^a^	41	Able to talk worries/fears	0.412^g^
59	Overall, how would you rate your care?	0.867^a^	42	Did they have the right documents?	0.644^g^
			53	Did GP/practice nurse did their best?	0.551^k^
*Factor 3—Diagnostic process*					
7	Were test results explained?	0.432^a^			
8	Did you know you could bring family member?	0.587^a^			
9	How do you feel about the way told Dx?	0.806^a^			
10	Did you understand the explanation of your Dx?	0.699^a^			
11	When told Dx, were you given written info about the type of cancer?	0.514^a^			
*Factor 4—Timeliness of investigations*					
2	Times you saw your GP before going to hospital about cancer	0.795^a^			
6	How did you feel about the length of time you had to wait for test?	0.794^a^			
*Factor 5—Aftercare and support*					
49	Did family/someone close get all info to help care for you at home?	0.472^a^	17	Were you given the name of a specialist nurse?	0.461^b^
55	Have you been given a care plan?	0.489^a^	20	Hospital gave enough info about support groups	0.782^d^
			21	Hospital staff discuss about impact of cancer on work/education	0.638^d^
			22	Hospital staff gave info about financial help	0.869^d^
			23	Hospital staff gave info on free prescriptions	0.757^d^
			50	During your tx were you given enough care and support from health or social services?	0.912^j^
			51	After your tx were you given enough care and support from health or social services?	1.045^j^
*Factor 6—Specialist nursing*					
			18	Was it easy to contact your specialist nurse?	0.819^c^
			19	Did the specialist nurse give answers you could understand?	0.802^c^
*Factor 7—Hospital stay*					
			29	Confidence and trust in doctors	0.481^e^
			31	Confidence and trust in nurses	0.914^e^
			32	In your opinion, were there enough nurses?	0.693^e^
			34	Enough privacy to discuss	0.586^e^
			36	Did they do everything to control pain?	0.664^e^
			37	Treated with respect and dignity	0.946^e^

^a^ From model with the 19 core questions applicable to all patients.

^b^ From five factor model with core questions and the question about access to clinical nurse specialists.

^c^ From six factor model with core questions and questions applicable to patients with access to specialist nurse.

^d^ From five factor model with core questions and questions applicable to patients with recent hospital care.

^e^ From six factor model with core questions and questions applicable to patients with recent hospital stay.

^f^ From six factor model with core questions and questions applicable to patients with recent hospital stay.

^g^ From five factor model with core questions and questions applicable to patients with recent outpatient or day case appointments.

^h^ From five factor model with core questions and questions applicable to patients treated by radiotherapy.

^i^ From five factor model with core questions and questions applicable to patients treated by chemotherapy.

^j^ From five factor model with core questions and questions applicable to patients who received support from health and social care services.

^k^ From five factor model with core questions and question applicable to patients with recent outpatient appointments.

In CFA, these five and six factor models (Appendix 1, Table A3) were found to provide a good/acceptable fit to the data according to RMSEA and SRMR (RMSEA range 0.042–0.058 and SRMR range 0.028–0.056). The CFI and TLI statistics for these models were closed to achieving, or achieved, an acceptable fit (CFI range 0.931–0.954 and TLI range 0.919–0.944).

## 5 DISCUSSION

### 5.1 Summary of findings

We have applied exploratory and confirmatory factor analyses to the responses to the English CPES. We found that the core set of questions which applied to all patients, and many questions which applied only to a subset of patients were dominated by a single underlying factor (as indicated by factor eigenvalues > 1). However, this single factor did not provide a good description of the data (according to goodness‐of‐fit metrics), thus implying a more complex underlying structure. Visual inspection of scree plots implied that five underlying factors can describe the experiences of patients captured by the core questions applicable to all patients. These included: shared decision‐making; care coordination and administration; the diagnostic process; timeliness of investigations; and aftercare and support. Many questions applicable to specific subsets of patients also fitted within the latter five underlying domains, but additional factors were required for specialist nursing and for hospital stay. These domains of care provide a good description of the data (according to goodness‐of‐fit metrics). Furthermore, they largely fitted with the existing structure of the survey and, in light of the data presented here, represent a reasonable target for public reporting of data and performance improvement.

### 5.2 Comparisons with the literature

Previous work has examined the underlying structure of other nationwide patient experience surveys including HCAHPS and GPPS,^30^, ^31^ but this is the first time that this approach has been applied to an established nationwide experience of cancer patients. It has been long recognized that patient experience varies greatly by patient sociodemographic characteristics, including age, sex, socioeconomic status, and ethnicity.^32^, ^33^ Furthermore, for CPESs, cancer site/type is strongly associated with ratings of experience, above and beyond adjustment for other patient‐level variables.^32^, ^34^ Future work should address the question whether the underlying structure of CPES may vary by patient group.

### 5.3 Strengths and limitations

We used a large sample, which allowed for precise estimation of underlying factors. We have only used data from a single year, however the survey has been conducted seven times between 2010 and 2019. During this period, there have been only small changes made to the wording of survey items. However, the number and type of question have remained largely the same, and the overall structure has remained consistent with the same sections covering the various stages of the care pathway. Therefore, the findings are likely to be generalizable across survey waves.

We note that in general core questions relating to the different factors tend to be placed in close proximity to each other within the questionnaire. While this can be useful to the patient, it is possible that this proximity influenced the factor structure we observed. However, when we also considered the questions applicable to certain patient groups or care pathways we found similar questions loading on to the same factor which were placed at some distance within the questionnaire. This would not be expected if proximity was the driving force behind the observed factor and thus provides further support for the 5/6 factor structure we propose.

### 5.4 Implications

Our results support the current structure of the survey which in general covers the range of aspects of care and patient experience which are relevant to cancer patients. The survey seems to capture the experience of patient groups defined by different care pathways and services equally well. Furthermore, the results indicate that although factual questions (such as about participating in research, Q58) could be successfully included in care experience questionnaires, it is important to recognize that these do not, on the basis of results presented here, represent aspects of patient experience per se.

This analysis can be used as the basis of supporting the construct of a number of composite indicators to summarize hospital performance with respect to cancer patient experience. For example, such composites, might target organizational performance across aspects of care experience relating to the five underlying domains/factors identified (i.e., shared decision‐making; care coordination and administration; the diagnostic process; timeliness of investigations; and aftercare and support). Alongside consideration of the drivers of satisfaction with care, such composites may help users of the survey to more easily relate to study findings and prioritize bundles of actions and interventions targeting specific composite domains (we explore this further below). This could, in principle, help to increase the reliability of organizational‐level scores, which are known to represent a limitation of question‐based scores of the CPES survey, though this needs to be explored directly in further empirical research.

It is worth noting that while factor analysis can provide evidence about patterns of responses, it tells us little about the relative importance of the various aspects of care. If an overall summary score were to be derived, various weighting schemes could be applied. All questions or domains of care could be weighted equally, though this implies they have equal importance. Alternatively, policy‐based weights may be employed reflecting an external view of the importance of different domains of care. A third option is to employ a key drivers analysis which empirically examines the importance of survey items to survey responders using their associations with a global evaluation item. Such a key drivers analysis has been carried out for a number of surveys, including CPES.^28^, ^35^, ^36^, ^37^ Many of these use a selection of individual questions, but others use domain scores, which can be based on factor analyses.

The CPES is a survey with a relatively large number of questions. As such there may be some desire to shorten the questionnaire to reduce burden on responding patients. The high internal consistency of Factor 1 (shared decision‐making) and Factor 3 (diagnostic process) indicates the potential for item removal. In contrast, the low internal consistency of Factor 5 (aftercare and support) indicates that there may be benefit in additional questions in this area. While factor analysis can help to identify potential questions for removal (for example by identifying domains of experience survey by a large number of questions) it should be noted that factor analysis is not considered sufficient for such purposes.^22^, ^23^ First, removing a question from a survey could be detrimental to its content validity. Furthermore, weak loadings might be the result of sampling error, although this is unlikely to be an issue in our study context, given the large sample size. As a consequence, replication of factor analytic models is critical for scale development.

## 6 CONCLUSION

The underlying structure of the CPES corresponds to five major aspects of care experience and pathways of cancer patients. The findings support the current survey design, though they also provide potential options to guide survey redesign, and have potential to inform the way the survey findings might optimally be reported, and improvement efforts targeted.

## CONFLICT OF INTEREST

The authors report grants from MacMillan Cancer Support, during the conduct of the study. GA and GL have acted as academic consultants providing methodological advice to NHS England Insight team regarding the Cancer Patient Experience Survey during 2015‐17.

## ETHICS APPROVAL

This research used anonymized data that are available for bona fide research by the UK Data Archive. As such no ethics approval of such research is required.

## PATIENT CONSENT FOR PUBLICATION

This work is a secondary analysis of anonymous data collected as part of a national survey programme. Each participant was provided with a detailed patient information leaflet and return of a questionnaire implied consent.

## Data Availability

Data used in this study are available via the UK Data Archive, Study Number (SN) 8163.

## APPENDIX 1

**TABLE A1:** Classification of questions into 11 sets representing a specific patient group or care pathway

Synoptic form	Patient group number	Patient group name
Q17. Were you given the name of a CNS	1	Question excluded from core set due to analytic reasons
Q18. Was it easy to contact your CNS	2	Patients with access to specialist nurse
Q19. Did the CNS give answers you could understand?		
Q20. Hospital gave enough info about support groups	3	Patients with recent hospital care
Q21. Hospital staff discuss about impact of cancer on work/education		
Q22. Hospital staff gave info about financial help		
Q23. Hospital staff gave info on free prescriptions		
Q26. How operation had gone explained afterward	4	Patients with recent operation
Q28. Did doctors speak of you as if you were not there?	5	Patients with recent hospital stay
Q30. Were family members able to speak to doctor?		
Q33. Were you asked how you want to be called?		
Q35. Able to discuss worries/fears		
Q29. Confidence and trust in doctors		
Q31. Confidence and trust in nurses		
Q32. In your opinion, were there enough nurses?		
Q34. Enough privacy to discuss		
Q36. Did they do everything to control pain?		
Q37. Treated with respect and dignity		
Q38. Clear written information about what you should (not) do after discharge		
Q39. Were you told who to contact after leaving hospital if needed		
Q41. Able to talk worries/fears	6	Patients with recent outpatient or day case appointments
Q42. Did they have the right documents?		
Q44. Beforehand did you have all the information you needed for radiotx	7	Patients treated by radiotherapy
Q45. Were you given enough information about whether your radiotx was working?		
Q47. Beforehand did you have all the information you needed for chemotx	8	Patients treated by chemotherapy
Q48. Were you given enough information about whether your chemotx was working?		
Q50. During your tx were you given enough care/support from health/social services?	9	Patients who received support from health and social care services
Q51. After your tx were you given enough care/support from health/social services?		
Q53. Did GP/practice nurse did their best?	10	Patients accessing primary care post‐discharge
Q57. Overall, how do you feel about the length of time to wait for clinics and appointments?	11	Patients with recent outpatient appointments

**TABLE A2:** Cronbach's alpha for the five factors derived from the exploratory factor analysis model restricted to five factors and applied to the core set of 19 questions

	Cronbach's alpha coefficient	Range of alphas if individual items deleted
Factor 1: Shared decision‐making (five items)	0.90	0.87–0.89
Factor 2: Care coordination and administration (four items)	0.74	0.61–0.79
Factor 3: Diagnostic process (five items)	0.81	0.76–0.79
Factor 4: Timeliness of investigations (two items)	0.79	—
Factor 5: Aftercare and support (two items)	0.60	—

**TABLE A3:** Goodness‐of‐fit statistics for each model

Model	One/two factors				Five/six factors			
	RMSEA	CFI	TLI	SRMR	RMSEA	CFI	TLI	SRMR
Core	0.081	0.836	0.814	0.054	0.045	0.9540	0.944	0.0290
Core + CNS name	0.081	0.836	0.814	0.054	0.044	0.9510	0.941	0.0290
Core + CNS	0.079	0.8150	0.793	0.0550	0.04	0.953	0.943	0.028
Core + support for cancer patients	0.073	0.872	0.857	0.049	0.046	0.946	0.938	0.033
Core + operation	0.079	0.838	0.818	0.053	0.045	0.954	0.944	0.029
Core + overnight stay	0.058	0.882	0.871	0.046	0.042	0.935	0.926	0.034
Core + outpatient/day case	0.075	0.837	0.818	0.052	0.045	0.946	0.936	0.031
Core + radiotx	0.079	0.824	0.804	0.056	0.047	0.941	0.93	0.034
Core + chemotx	0.075	0.844	0.826	0.052	0.045	0.947	0.937	0.031
Core + health/social services	0.095	0.837	0.814	0.073	0.058	0.931	0.919	0.056
Core + care from GP	0.082	0.826	0.804	0.055	0.049	0.943	0.931	0.031
Core + overall care	0.078	0.832	0.811	0.053	0.045	0.954	0.944	0.029

Abbreviations: CFI, comparative fit index; RMSEA, root mean square error of approximation.

## References

[1] Locock L , Graham C , King J , et al. Health services and delivery research. In Understanding How Front‐Line Staff Use Patient Experience Data for Service Improvement: An Exploratory Case Study Evaluation. NIHR Journals Library; 2020. 32182003

[2] Llanwarne NR , Abel GA , Elliott MN , et al. Relationship between clinical quality and patient experience: analysis of data from the English Quality and Outcomes Framework and the National GP Patient Survey. Ann Fam Med. 2013;11(5):467‐472. 24019279 10.1370/afm.1514 PMC3767716

[3] Campbell J . Patients' experience of primary care: James Mackenzie Lecture 2017. Br J Gen Pract. 2019;69(678):38‐39. 30591612 10.3399/bjgp19X700601 PMC6301360

[4] Care Quality Commission . Adult inpatient survey 2018. Accessed November 11, 2021. https://nhssurveys.org/surveys/survey/02‐adults‐inpatients/year/2018/

[5] Centers for Medicare and Medicaid Services Consumer Assessment of Healthcare Providers and Systems (CAHPS). Accessed September 07, 2018. https://www.cms.gov/Research‐Statistics‐Data‐and‐Systems/Research/CAHPS/

[6] Agency for Healthcare Research and Quality . CAHPS Cancer Care Survey. 2017. Accessed December 02, 2021. https://www.ahrq.gov/cahps/surveys‐guidance/cancer/index.html

[7] NHS England . GP Patient Survey. 2016. Accessed December 02, 2021. https://www.england.nhs.uk/statistics/2016/07/07/gp‐patient‐survey‐2015‐16/

[8] Quality Health . National Cancer Experience Survey 2016. Accessed November 11, 2021. https://www.gov.uk/government/statistics/national‐cancer‐patient‐experience‐survey‐2016‐national‐data

[9] Campbell J , Smith P , Nissen S , Bower P , Elliott M , Roland M . The GP Patient Survey for use in primary care in the National Health Service in the UK—development and psychometric characteristics. BMC Family Practice. 2009;10(1):57. 19698140 10.1186/1471-2296-10-57 PMC2736918

[10] Marshall GN , Morales LS , Elliott M , Spritzer K , Hays RD . Confirmatory factor analysis of the Consumer Assessment of Health Plans Study (CAHPS) 1.0 core survey. Psychol Assess. 2001;13(2):216‐229. 11433796 10.1037//1040-3590.13.2.216 PMC1781360

[11] Hays RD , Martino S , Brown JA , et al. Evaluation of a care coordination measure for the Consumer Assessment of Healthcare Providers and Systems (CAHPS®) medicare survey. Med Care Res Rev. 2014;71(2):192‐202. 24227813 10.1177/1077558713508205 PMC3959996

[12] Lee Hargraves J , Hays RD , Cleary PD . Psychometric properties of the consumer assessment of health plans study (CAHPS®) 2.0 adult core survey. Health Serv Res. 2003;38(6p1):1509‐1528. 14727785 10.1111/j.1475-6773.2003.00190.x PMC1360961

[13] Solomon LS , Hays RD , Zaslavsky AM , Ding L , Cleary PD . Psychometric properties of a group‐level Consumer Assessment of Health Plans Study (CAHPS) instrument. Med Care. 2005;43(1):53‐60. 15626934

[14] O'Malley AJ , Zaslavsky AM , Hays RD , Hepner KA , Keller S , Cleary PD . Exploratory factor analyses of the CAHPS® hospital pilot survey responses across and within medical, surgical, and obstetric services. Health Serv Res. 2005;40(6p2):2078‐2095. 16316439 10.1111/j.1475-6773.2005.00471.x PMC1361242

[15] Dyer N , Sorra JS , Smith SA , Cleary P , Hays R . Psychometric properties of the Consumer Assessment of Healthcare Providers and Systems (CAHPS®) clinician and group adult visit survey. Med Care. 2012;50(Suppl):S28‐S34. 23064274 10.1097/MLR.0b013e31826cbc0d PMC3480671

[16] Ramsay J , Campbell JL , Schroter S , Green J , Roland M . The General Practice Assessment Survey (GPAS): tests of data quality and measurement properties. Fam Pract. 2000;17(5):372‐379. 11021894 10.1093/fampra/17.5.372

[17] Quality Health . National Cancer Patient Experience Survey 2015. National Results Summary. Accessed December 02, 2021. https://www.ncpes.co.uk/wp‐content/uploads/2020/06/National‐Cancer‐Patient‐Experience‐Survey‐2015‐National‐Report.pdf

[18] Shi D , Lee T , Fairchild AJ , Maydeu‐Olivares A . Fitting ordinal factor analysis models with missing data: a comparison between pairwise deletion and multiple imputation. Educ Psychol Measur. 2020;80(1):41‐66. 31933492 10.1177/0013164419845039 PMC6943991

[19] Revelle W . psych: procedures for psychological, psychometric, and personality research. In R package version 1812. 2018. Accessed November 11, 2021. https://cran.r‐project.org/web/packages/psych/index.html

[20] Kaiser HF . The application of electronic computers to factor analysis. Educ Psychol Measur. 1960;20(1):141‐151.

[21] Hayton JC , Allen DG , Scarpello V . Factor retention decisions in exploratory factor analysis: a tutorial on parallel analysis. Organ Res Methods. 2004;7(2):191‐205.

[22] Flora DB , Flake JK . The purpose and practice of exploratory and confirmatory factor analysis in psychological research: decisions for scale development and validation. Can J Behav Sci. 2017;49(2):78.

[23] Bandalos DL , Finney SJ . Exploratory and confirmatory. In Hancock GR , Stapleton LM , Mueller RO , eds. The Reviewer's Guide to Quantitative Methods in the Social Sciences New York. Taylor and Francis; 2018;93‐114.

[24] Howard MC . A review of exploratory factor analysis decisions and overview of current practices: what we are doing and how can we improve? Int J Hum‐Comput Int. 2016;32(1):51‐62.

[25] Hooper D , Coughlan J , Mullen MR . Structural equation modelling: guidelines for determining model fit. Electron J Bus Res Methods. 2008;6(1):53‐60.

[26] Lt HU , Bentler PM . Cutoff criteria for fit indexes in covariance structure analysis: conventional criteria versus new alternatives. Struct Equ Modeling. 1999;6(1):1–55.

[27] Rosseel Y . lavaan: an R package for structural equation modeling. J Stat Softw. 2012;48:1‐36.

[28] Gomez‐Cano M , Lyratzopoulos G , Abel GA . Patient experience drivers of overall satisfaction with care in cancer patients: evidence from responders to the English Cancer Patient Experience Survey. J Patient Exp. 2020;7(5):758–765. 33294612 10.1177/2374373519889435 PMC7705845

[29] R Core Team . R: A Language and Environment for Statistical Computing. R Foundation for Statistical Computing; 2019.

[30] Cefalu MS , Elliott MN , Setodji CM , Cleary PD , Hays RD . Hospital quality indicators are not unidimensional: a reanalysis of Lieberthal and Comer. Health Serv Res. 2019;54(2):502‐508. 30259508 10.1111/1475-6773.13056 PMC6407350

[31] Campbell J , Smith P , Nissen S , Bower P , Elliott M , Roland M . The GP Patient Survey for use in primary care in the National Health Service in the UK—development and psychometric characteristics. BMC Family Practice. 2009;10(1):57. 19698140 10.1186/1471-2296-10-57 PMC2736918

[32] Abel G , Saunders C , Lyratzopoulos G . General Practice Profiles for Cancer: how variable are the practices and can we reliably judge their diagnostic performance? Eur J Cancer Care. 2015;24:72.

[33] Lyratzopoulos G , Elliott M , Barbiere JM , et al. Understanding ethnic and other socio‐demographic differences in patient experience of primary care: evidence from the English General Practice Patient Survey. BMJ Qual Saf. 2012;21(1):21‐29. 10.1136/bmjqs-2011-000088 PMC3240774 21900695

[34] El Turabi A , Abel GA , Roland M , Lyratzopoulos G . Variation in reported experience of involvement in cancer treatment decision making: evidence from the National Cancer Patient Experience Survey. Br J Cancer. 2013;109:780‐787. 23807170 10.1038/bjc.2013.316 PMC3738115

[35] Quigley DD , Elliott MN , Farley DO , Burkhart Q , Skootsky SA , Hays RD . Specialties differ in which aspects of doctor communication predict overall physician ratings. J Gen Intern Med. 2014;29(3):447‐454. 24163151 10.1007/s11606-013-2663-2 PMC3930786

[36] Collins RL , Haas A , Haviland AM , Elliott MN . What matters most to whom. Med Care. 2017;55(11):940‐947. 28930888 10.1097/MLR.0000000000000804

[37] Elliott MN , Kanouse DE , Edwards CA , Hilborne LH . Components of care vary in importance for overall patient‐reported experience by type of hospitalization. Med Care. 2009;47(8):842‐849. 19584764 10.1097/MLR.0b013e318197b22a

